# Sex-dependent effect of socioeconomic status on cardiovascular event risk in a population-based cohort of patients with type 2 diabetes

**DOI:** 10.1093/eurpub/ckae048

**Published:** 2024-03-14

**Authors:** Mónica Enguita-Germán, Ibai Tamayo, Julián Librero, Asier Ballesteros-Domínguez, Ignacio Oscoz-Villanueva, Arkaitz Galbete, Laura Arnedo, Koldo Cambra, Javier Gorricho, Conchi Moreno-Iribas, Eduardo Millán-Ortuondo, Berta Ibáñez-Beroiz

**Affiliations:** Unidad de Metodología, Navarrabiomed-HUN-UPNA, Pamplona, Spain; Instituto de Investigación Sanitaria de Navarra (IdiSNA), Pamplona, Spain; Red de Investigación en Cronicidad, Atención Primaria y Promoción de la Salud (RICAPPS), Pamplona, Spain; Unidad de Metodología, Navarrabiomed-HUN-UPNA, Pamplona, Spain; Instituto de Investigación Sanitaria de Navarra (IdiSNA), Pamplona, Spain; Red de Investigación en Cronicidad, Atención Primaria y Promoción de la Salud (RICAPPS), Pamplona, Spain; Unidad de Metodología, Navarrabiomed-HUN-UPNA, Pamplona, Spain; Red de Investigación en Cronicidad, Atención Primaria y Promoción de la Salud (RICAPPS), Pamplona, Spain; Unidad de Metodología, Navarrabiomed-HUN-UPNA, Pamplona, Spain; Instituto de Investigación Sanitaria de Navarra (IdiSNA), Pamplona, Spain; Red de Investigación en Cronicidad, Atención Primaria y Promoción de la Salud (RICAPPS), Pamplona, Spain; Unidad de Metodología, Navarrabiomed-HUN-UPNA, Pamplona, Spain; Instituto de Investigación Sanitaria de Navarra (IdiSNA), Pamplona, Spain; Red de Investigación en Cronicidad, Atención Primaria y Promoción de la Salud (RICAPPS), Pamplona, Spain; Unidad de Metodología, Navarrabiomed-HUN-UPNA, Pamplona, Spain; Instituto de Investigación Sanitaria de Navarra (IdiSNA), Pamplona, Spain; Red de Investigación en Cronicidad, Atención Primaria y Promoción de la Salud (RICAPPS), Pamplona, Spain; Departamento de Estadística, UPNA, Pamplona, Spain; Instituto de Investigación Sanitaria de Navarra (IdiSNA), Pamplona, Spain; Dirección de Salud Pública y Adicciones, Departamento de Sanidad del Gobierno Vasco, Vitoria-Gasteiz, Spain; Servicio de Evaluación y Difusión de resultados en Salud, Servicio Navarro de Salud (SNS-O), Pamplona, Spain; Instituto de Investigación Sanitaria de Navarra (IdiSNA), Pamplona, Spain; Consorcio de Investigación Biomédica en Red de Epidemiología y Salud Pública (CIBERESP), Madrid, Spain. Instituto de Salud Pública, Pamplona, Spain; Osakidetza-Servicio Vasco de Salud, Bilbao, Spain; Unidad de Metodología, Navarrabiomed-HUN-UPNA, Pamplona, Spain; Instituto de Investigación Sanitaria de Navarra (IdiSNA), Pamplona, Spain; Red de Investigación en Cronicidad, Atención Primaria y Promoción de la Salud (RICAPPS), Pamplona, Spain

## Abstract

**Background:**

Socioeconomic status (SES) factors often result in profound health inequalities among populations, and their impact may differ between sexes. The aim of this study was to estimate and compare the effect of socioeconomic status indicators on incident cardiovascular disease (CVD)-related events among males and females with type 2 diabetes (T2D).

**Methods:**

A population-based cohort from a southern European region including 24,650 patients with T2D was followed for five years. The sex-specific associations between SES indicators and the first occurring CVD event were modeled using multivariate Fine-Gray competing risk models. Coronary Heart Disease (CHD) and stroke were considered secondary outcomes.

**Results:**

Patients without a formal education had a significantly higher risk of CVD than those with a high school or university education, with adjusted hazard ratios (HRs) equal to 1.24 (95%CI: 1.09–1.41) for males and 1.50 (95%CI: 1.09–2.06) for females. Patients with <18 000€ income had also higher CVD risk than those with ≥18 000€, with HRs equal to 1.44 (95%CI: 1.29–1.59) for males and 1.42 (95%CI: 1.26–1.60) for females. Being immigrant showed a HR equal to 0.81 (95%CI: 0.66–0.99) for males and 1.13 (95%CI: 0.68–1.87) for females. Similar results were observed for stroke, but differed for CHD when income is used, which had higher effect in females.

**Conclusion:**

Socioeconomic inequalities in CVD outcomes are present among T2D patients, and their magnitude for educational attainment is sex-dependent, being higher in females, suggesting the need to consider them when designing tailored primary prevention and management strategies.

## Introduction

Type 2 diabetes (T2D) prevalence worldwide is approximately 10.5%, and caused 6.7 million deaths in 2021, representing 9–11% of total health expenditures. Both the prevalence and associated costs are rising, and it is projected that 12.2% of the world population will have diabetes by 2045, with an associated cost of 1054 billion USD.^1^^,^^2^

Among patients with T2D, cardiovascular disease (CVD) represents the most common cause of morbidity and mortality.^3^ Early detection and effective management of T2D through periodic medical controls and medication when needed, together with education on self-care and adoption of healthy lifestyles, can prevent or delay the worsening of the disease and the occurrence of major CVD complications and their related health costs.^4^^,^^5^ Hence, the early identification and management of people with a disproportionately high risk of developing diabetes-related complications could mitigate the impact of this crucial worldwide health challenge.

Socioeconomic factors often result in profound health inequalities among populations. Strategies to achieve health and gender equity are on the former and current European agenda,^6^ but inadmissible inequalities persist. Income, educational attainment, employment status, and marital status have been related to CVD.^7–10^ Sex-specific inequalities, in terms of contextual macro level opportunities, and therefore, in the individual education level, occupations, financial independence and family responsibilities, may lead to sex-disparities in the magnitude and direction of the impact of certain SES factors on health outcomes.^11–14^ Further, these disparities could also differ when considering different CVD outcomes, such as coronary heart diseases (CHD) or stroke.^12^

A considerable body of evidence demonstrates that T2D complications are substantially higher among the most economically disadvantaged people. A recent scientific revision about social determinants of health in diabetes proposed a list of research topics, including observational and intervention studies to better understand and intervene on these determinants as root causes of diabetes disparities.^15^

Among T2D patients, some studies have shown an association between low individual SES and CVD risk factors,^16^^,^^17^ but studies on the relationship between individual SES and CVD outcomes are still scarce,^18–20^ particularly those that stratify by sex.^21^^,^^22^ Since it is well established that the gender differences on cardiovascular CVD mortality risk depends on the presence of diabetes,^23^ it is possible that having this disease also implies differences in the impact of SES on CVD risk between both sexes. Most of the prior evidence did not excluded patients with the previous CVD events,^18–21^ did not differentiate between CHD and stroke,^18–20^ or were not focused on contemporary populations.^18–21^ Additionally, none of these previous works accounted for competing risks. The aim of this study was to determine the differential effect of several individual SES markers (education level, mean income or immigrant status) on fatal and nonfatal incident CHD, stroke and CVD events among males and females with T2D in a population-based cohort from a southern European region with 5 years of follow-up.

## Methods

### Study population

Patients included in this retrospective population-based cohort study were residents of Navarra autonomous region in northern Spain, where citizens have free access of care by the Regional Health Service of Navarra-Osasunbidea, which is part of the National Health System of Spain. In 2021, 0.24% of the Navarra population had exclusively private health insurance.^24^

The data used belonged to the population-based CARDIANA cohort, which contains anonymized patient-level information on socioeconomic variables, medical history records, relevant CVD risk factors and lifestyle-related variables of all patients with T1D (*n* = 1077) and all patients with T2D (*n* = 33 842) in Navarra (see cohort profile for more details).^25^ For the current study, we included males and females with prevalent T2D without a history of the previous CVD (codes K74, K75, K76, K77, K89, K90, and K91 in the International Classification of Primary Care, version 2 [ICPC-2]) at baseline and with no missing data on individual income, level of education, immigrant status or mean income of the area. Participants were followed for 5 years, from January 1, 2012, to December 31, 2016. The study protocol was favorably evaluated by the Ethics Committee of Clinical Research of Navarra (Project 2015/111, 20 October 2015), and in the CONCEPT-Project framework (Project 97/2019, 28 August, 2019).

### Exposure

Sex and three different patient-level SES-related variables were evaluated: level of education, yearly individual income and immigrant status. The level of education was based on information extracted from the population register within the year before baseline, while individual income and immigrant status were based on the values as they appeared in the clinical records at baseline. The annual mean income of the census tract of the year before baseline was also taken into account in the analyses as an area-level clustering variable to adjust for contextual economic inequalities. The original level of education variable had four categories, namely, none, primary school, high school and university studies, but the last two were merged for analysis purposes due to the scarcity of patients with university studies, especially among females. The household income was obtained from the Spanish drug cost-sharing scheme established in 2012. This scheme creates copayment categories combining declared annual income tax (with cut-off points of 18 000 and 100 000 euros) and pensioner status (retirement from work or disability). Given the small proportion of people that exceeds 100 000 euros, income was categorized into two categories: income ≥18 000€ and <18 000€. Immigrant status was considered when the country of origin of the patient was other than Spain. Finally, the annual mean income of the census tract was categorized into quintiles. Patients with no data for these variables were not included in the analyses.

### Outcomes

The principal endpoint was defined as the first occurring fatal or nonfatal CVD event during the 5 year follow-up. A CVD event was considered to occur when a CVD diagnosis was recorded in the mortality registry or in the Minimum Basic DataSet (MBDS) as defined in the SCORE2 study^26^ (see Supplementary table S1). Additionally, two secondary endpoints were considered, CHD (fatal; ICD9-codes 410–414 and ICD10-codes I20–25, and non-fatal; ICD9-codes 410 and ICD10-codes I21–23) and stroke (fatal or non-fatal; ICD9-codes 431–438, excluding 432.1, 437.3 and 437.4 and ICD10-codes I61, I63–69 excluding I67.1, I68.2 and I67.5).

### Confounders

Several known CVD risk factors were taken into account in the analysis: age (four categories), time since T2D diagnosis, the presence of other comorbidities (using the abbreviated version of the Charlson Comorbidity Index, aCharlson),^27^^,^^28^ smoking status and physical activity. The aCharlson score assigned one point for each of the following comorbidities: CVD, diabetes, heart failure, chronic obstructive pulmonary disease (COPD), dementia and peripheral artery disease and two points for chronic renal failure (CRF) and cancer. In our sample, all patients had diabetes but none had previous CVD, thus the score ranged from 1 to 9 points. Lifestyle information was obtained from primary care records, and it was parameterized using regular expressions to convert text information into the final categories. Baseline values for lifestyle factors were considered valid if recorded within a 5-year time window before the start date, and the closest value to this date was selected.

### Statistical analysis

Baseline characteristics of the total cohort and by sex were summarized using descriptive measures, and the cumulative incidence of CVD events was calculated and visually presented using sex-specific Kaplan–Meier curves by each SES-related variable. Patients were right censored if it was notified that they moved to another region or died by any non-CVD cause. To evaluate the sex-specific effect of each SES variable on CVD events, we stratified the cohort by sex and fitted Fine-Gray competing risk models considering the non-CVD deaths as competing events. The main model (named ‘min-adjusted’) included one SES indicator (level of education, income or immigrant status) as exposure variable, the annual mean income of the area as a clustering variable and age, duration of diabetes and aCharlson score as covariates. The ‘max-adjusted’ model added smoking status and physical activity to the min-adjusted model. As both life-style factors contained missing data, an additional category named ‘NA’ was included in order to maintain the complete sample in the ‘max-adjusted’ models. Finally, a model that included all three SES indicators was also fitted in order to determine the independent contribution of each indicator while adjusting for the others. Results were presented as hazard ratios (HRs) with their 95%CIs. The min-adjusted models that included each SES indicator separately were represented graphically in a sex-combined forest plot. The proportionality assumption was evaluated by testing and plotting the correlation between the corresponding scaled Schoenfeld residuals and time. To compare the magnitude of effects between sexes directly, we estimated female-to-male ratios of HRs (RRRs) for each SES indicator, as previously described.^12^ The sex-specific effect of each SES variable on stroke and CHD separately was assessed using the same methods than for the global CVD outcome.

## Results

The study cohort consisted of all 24 650 T2D patients who had no previous history of CVD (76.2%) and that had valid information for all SES variables (95.6%). Complete information regarding smoking status and physical activity were available for the 75% of the individuals. The baseline characteristics of these study participants are shown in table 1. The mean age of the total cohort was 67.6 years, and they had a median time since T2D diagnosis of 7.0 years. More than half were males (54.5%), 14.6% were current smokers, 8.7% were physically inactive and 29.4% had more than two points in the aCharlson score. With respect to SES variables, 32.4% had no formal education, 69.7% belonged to the <18 000€ income category and 5.2% were immigrants. Compared with men, women were five years older and had a higher probability of being physically inactive but lower probability of being smokers or having other comorbidities. The proportion of females without formal education or belonging to the 18 000€ income category was 12% and 23% higher than that for males, respectively. The proportion of immigrants was similar for both sexes, but the continent of origin differed slightly. Baseline characteristics of the total cohort and stratified by sex were also described according to education level, income and immigrant status (see Supplementary tables S2–S4).

**Table 1:** Baseline characteristics according to the sex in subjects with prevalent type 2 diabetes

Variable	Levels	Total cohort	Males	Females
*N* (%)		24 650	13 426 (55.4)	11 224 (44.6)
Patient-level characteristics				
Age	Mean (SD)	67.6 (12.8)	65.2 (12.1)	70.4 (13.0)
Age group	<60 years	6375 (25.9)	4179 (31.1)	2196 (19.6)
	60–69 years	6996 (28.4)	4280 (31.9)	2716 (24.2)
	70–79 years	6608 (26.8)	3289 (24.5)	3319 (29.6)
	≥80 years	4671 (18.9)	1678 (12.5)	2993 (26.7)
Sex	Male	13 426 (54.5)	13 426 (100.0)	0 (0.0)
	Female	11 224 (45.5)	0 (0.0)	11 224 (100.0)
Smoking status	Non-smoker	12 425 (50.4)	4270 (31.8)	8155 (72.7)
	Ex-smoker	4505 (18.3)	3807 (28.4)	698 (6.2)
	Smoker	4131 (16.8)	3222 (24.0)	909 (8.1)
	NA	3589 (14.6)	2127 (15.8)	1462 (13.0)
Physical activity	Inactive	2056 (8.3)	835 (6.2)	1221 (10.9)
	Partially active	5829 (23.6)	2663 (19.8)	3166 (28.2)
	Active	11 418 (46.3)	6812 (50.7)	4606 (41.0)
	NA	5347 (21.7)	3116 (23.2)	2231 (19.9)
Duration T2D	Median (IQR)	7.0 [4.0, 11.0]	7.0 [3.0, 10.0]	7.0 [4.0, 11.0]
aCharlson	1–2	17 412 (70.6)	9184 (68.4)	8228 (73.3)
group	≥3	7238 (29.4)	4242 (31.6)	2996 (26.7)
Patient-level SES characteristics				
Education level	Without studies	7978 (32.4)	3611 (26.9)	4367 (38.9)
	Primary School	13 064 (53.0)	7183 (53.5)	5881 (52.4)
	HS-University	3608 (14.6)	2632 (19.6)	976 (8.7)
Income	<18 000€	17 181 (69.7)	7958 (59.3)	9223 (82.2)
	≥18 000€	7469 (30.3)	5468 (40.7)	2001 (17.8)
Immigrant	No	23 361 (94.8)	12 798 (95.3)	10 563 (94.1)
	Yes	1289 (5.2)	628 (4.7)	661 (5.9)
Continent of origin for immigrants	Spain	23 361 (94.8)	12 798 (95.3)	10 563 (94.1)
	Africa	244 (1.0)	148 (1.1)	96 (0.9)
	America	587 (2.4)	233 (1.7)	354 (3.2)
	Asia-Australia	44 (0.2)	17 (0.1)	27 (0.2)
	Others Europe	414 (1.7)	230 (1.7)	184 (1.6)

Note: SES, socioeconomic status; T2D, diabetes mellitus; aCharlson, abbreviated Charlson comorbidity; HS-University, high school–University; SD, standard deviation; IQR, interquartile range.

**Figure 1:**
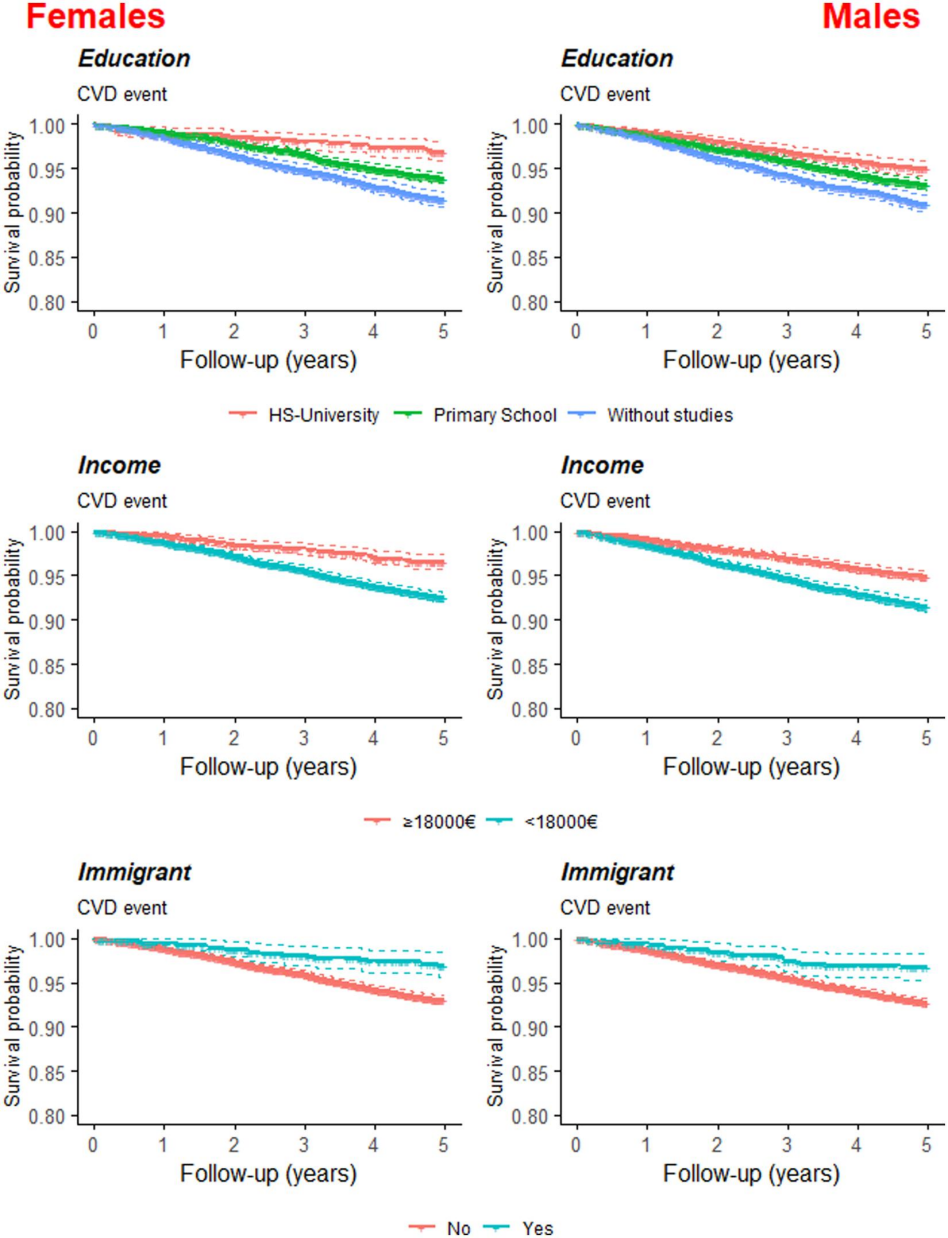
Kaplan Meier curves by SES-related variables in males and females with Type 2 diabetes

The mean follow-up of the total cohort was 4.5 years, with a total of 111 108 person-years. Among all included participants, 1588 fatal and nonfatal CVD cases were reported, 884 among males and 704 among females. Considering all-cause mortality, 3168 patients died, 1651 males and 1517 females. A total of 201 (0.81%) were right-censored for having moved to another region. The cumulative incidence in the total cohort was 6.9 (95%CI: 6.6–7.2) for CVD (males: 7.1; 95%CI: 6.6–7.5; females: 6.7; 95%CI: 6.3–7.2), 2.2 (95%CI: 2–2.4) for CHD (males: 2.5; 95%CI: 2.2–2.8; females: 1.8; 95%CI: 1.5–2.0) and 4.0 (95%CI: 3.8–4.3) for stroke (males: 3.9; 95%CI: 3.6–4.3; females: 4.1; 95%CI: 3.7–4.5). Kaplan–Meier curves for all individual SES indicators for CVD are shown in figure. 1. Patients in the lowest education level and income category had a higher cumulative CVD incidence than those in higher categories, whereas immigrant patients had a lower cumulative CVD incidence than native patients.

**Figure 2:**
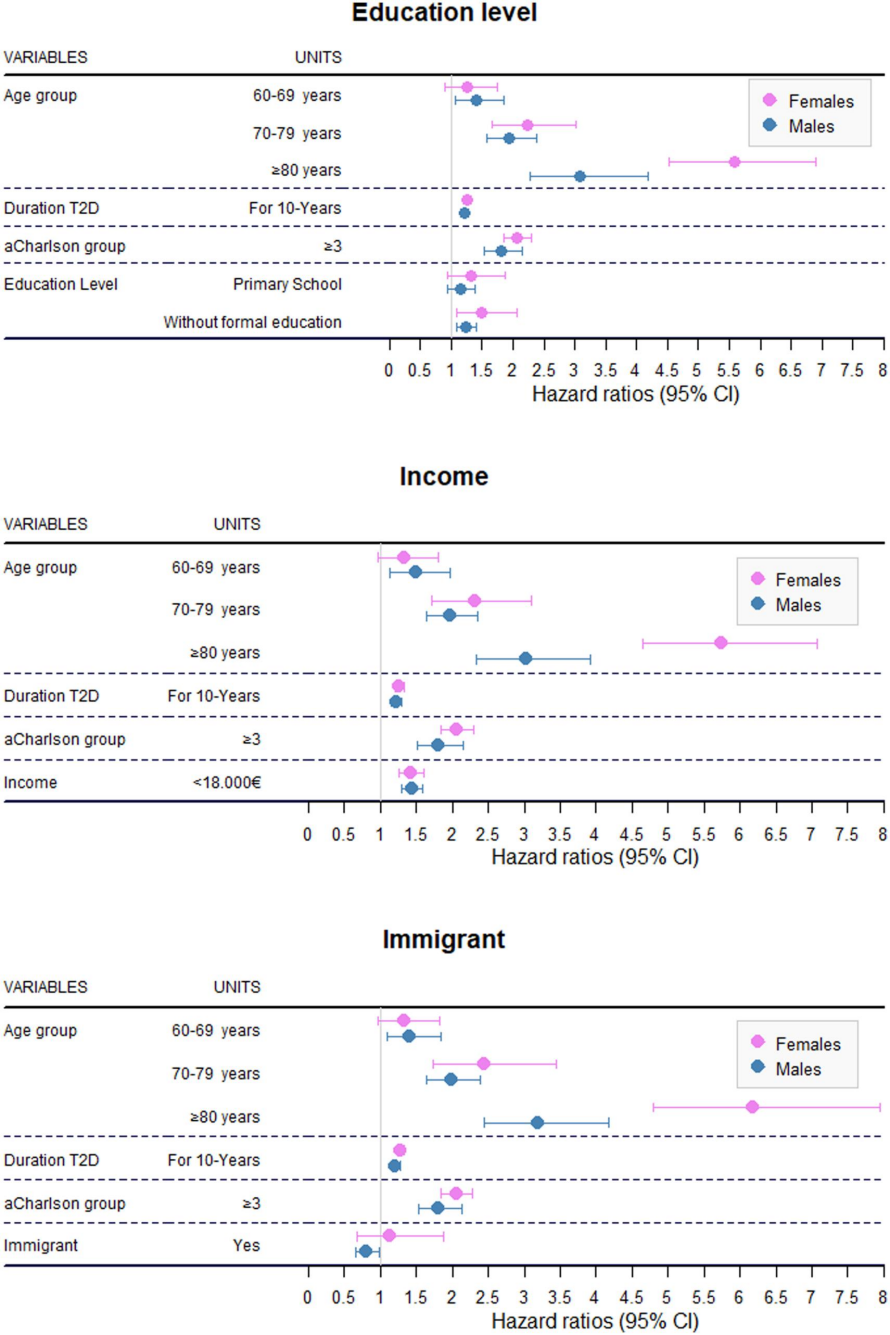
Adjusted hazard ratios for sex-stratified Fine-Gray min-adjusted models of SES variables on CVD event risk in patients with Type 2 Diabetes

The adjusted HRs of the sex-stratified multivariate regressions of education level, income and immigrant status are summarized in table 2. Detailed information on min-adjusted models is given in Supplementary tables S5–S7 and is graphically plotted for CVD in figure 2. All covariates age, diabetes duration and aCharlson score were significant CVD risk factors, all with associated HRs slightly higher among females. Regarding SES variables, females with no formal education had a significantly higher CVD risk than those with high school or university studies, with an adjusted HR equal to 1.50 (95%CI: 1.09–2.06), whereas in males, a significant but more modest effect was observed (HR = 1.24; 95%CI: 1.09–1.41), giving a RRR = 1.21 (95%CI: 0.86–1.71). The magnitude of RRR was similar for stroke (1.21; 95%CI: 0.74–1.97) and lower for CHD (0.98; 95%CI: 0.48–1.98). With regard to income, both females and males in the <18000€ income category had a higher CVD risk than those in the ≥18 000€ income category, with adjusted HRs equal to 1.42 (95%CI: 1.26–1.60) for females and 1.44 (95%CI: 1.29–1.59) for males, and a RRR = 0.99 (95%CI: 0.84–1.16). The magnitude of the effect of income was higher for stroke among males and for CHD among females. The RRR was 0.81 (95%CI: 0.58–1.14) for stroke and 1.63 (95%CI: 0.82–3.25) for CHD. Finally, being immigrant showed a protective effect among males, with HR = 0.81 (95%CI: 0.66–0.99), and female-to-male RRR = 1.4 (95%CI: 0.81–2.41). When considering separately stroke and CHD, the low number of events in immigrant population led to imprecise estimations, with RRR =1.84 (95%CI: 0.73-4.64) for stroke and 0.82 (95%CI: 0.44–1.52) for CHD.

**Table 2:** Multivariate Fine-Gray min-adjusted model HRs (95%CI) of CVD according to the SES indicators among males and females with type 2 diabetes

		Males	Females	Females/males
	Categories	HR (95CI, *P*-value)	HR (95CI, *P*-value)	RRR (95CI)
Education level				
CVD	HS-University	Reference	Reference	
	Primary school	1.15 (0.95–1.38, *P* = 0.160)	1.33 (0.94–1.88, *P* = 0.100)	1.16 (0.78–1.71)
	Without studies	1.24 (1.09–1.41, *P* = 0.001)	1.50 (1.09–2.06, *P* = 0.012)	1.21 (0.86–1.71)
Stroke	HS-University	Reference	Reference	
	Primary school	1.26 (1.07–1.49, *P* = 0.005)	1.45 (1.03–2.04, *P* = 0.031)	1.15 (0.79–1.68)
	Without studies	1.21 (0.97–1.51, *P* = 0.090)	1.46 (0.94–2.26, *P* = 0.088)	1.21 (0.74–1.97)
CHD	HS-University	Reference	Reference	
	Primary School	1.02 (0.83–1.27, *P* = 0.840)	1.00 (0.56–1.76, *P* = 0.990)	0.98 (0.53–1.81)
	Without studies	1.28 (1.12–1.45, *P* < 0.001)	1.25 (0.62–2.49, *P* = 0.530)	0.98 (0.48–1.98)
Income				
CVD	≥18 000€	Reference	Reference	
	<18 000€	1.44 (1.29–1.59, *P* < 0.001)	1.42 (1.26–1.60, *P* = 0.000)	0.99 (0.84–1.16)
Stroke	≥18 000€	Reference	Reference	
	<18 000€	1.53 (1.39–1.68, *P* < 0.001)	1.24 (0.90–1.72, *P* = 0.180)	0.81 (0.58–1.14)
CHD	≥18 000€	Reference	Reference	
	<18 000€	1.34 (1.04–1.72, *P* = 0.022)	2.19 (1.15–4.14, *P* = 0.016)	1.63 (0.82–3.25)
Immigrant status				
CVD	No	Reference	Reference	
	Yes	0.81 (0.66–0.99, *P* = 0.038)	1.13 (0.68–1.87, *P* = 0.640)	1.4 (0.81–2.41)
Stroke	No	Reference	Reference	
	Yes	0.83 (0.47–1.46, *P* = 0.520)	1.53 (0.74–3.17, *P* = 0.250)	1.84 (0.73–4.64)
CHD	No	Reference	Reference	
	Yes	0.99 (0.67–1.47, *P* = 0.970)	0.81 (0.50–1.30, *P* = 0.380)	0.82 (0.44–1.52)

Note: HS-University, High School – University. The min-adjusted model included the individual-level SES indicator (level of education, income or immigrant status), the annual mean income of the area as a clustering variable and age, duration of diabetes and aCharlson score as potential confounders.

The analysis of the max-adjusted models that included smoking status and physical activity variables, did not substantially change the SES-associated effects for any of the outcomes (see the last rows of Supplementary tables S5–S7). Complementary analysis including additional risk factors for model adjustment (hypertension, dyslipidemia or alcohol) also yielded similar results (data not shown). Finally, results obtained when including all SES indicators together in the same model were consistent with those obtained in the individual models (see Supplementary table S8) regardless the model covariates (min or max-adjusted) or the outcome.

## Discussion

This population-based cohort study showed that education level, income and immigrant status have an important impact on CVD risk among patients with T2D and its magnitude is slightly sex-dependent. Having a low education level increased this risk in both sexes, with a higher magnitude among females for CVD and stroke. Having low income increased the risk of CVD similarly regardless sex, but seems to have higher impact in males for stroke and in females for CHD. Being male and immigrant was associated with a lower risk of CVD compared with being native. These results remained consistent after adjustment for potential mediators, such as smoking status, physical activity or other SES indicators, suggesting the presence of gender differences in the SES related health inequalities in T2D patients.

Our findings regarding the inverse association between SES and CVD risk are consistent with most studies on socioeconomic inequalities in health that identify their presence worldwide for many health outcomes, including CVD and mortality.^8–10^^,^^29^ Recent evidence suggests that the association between SES indicators and CVD mortality can be comparable in strength to that of traditional CVD risk factors.^30^ For the education indicator, the HR estimates in our study ranged from 1.2–1.5, in line with other studies conducted in patients with diabetes,^19^^,^^20^ but slightly lower than those found in general population, which ranged from 1.5 to 1.7.^14^ Regarding income, our results gave HR estimates about 1.4, which agrees with the previous literature in both patients with diabetes^19^ and in general population.^14^

When we focused on comparing SES effects on CVD risk among males and females, we found that the impact of having low vs. high education level on CVD event risk was higher among females, with a RRR equal to 1.21 (95%CI: 0.86–1.71), and remained virtually the same when including lifestyle factors and when all SES indicators were modeled together. This magnitude is in line with the obtained in a meta-analysis conducted in 2016 for general population (RRR = 1.23; 95%CI: 1.03, 1.48)^12^ but higher than the RRR calculated from the HR reported in the most recent meta-analysis on 2023 (RRR = 1.11; 95%CI: 0.88, 1.39).^14^ Hence, if we consider the most recent evidence, then we could hypothesize that sex-dependent SES inequalities may be accentuated in patient with diabetes.

The protective effect of having higher educational attainment may be stronger in females than in males because, in most cases, they were the housekeepers, and to have higher educational level could have had a higher impact on overall familial well-being through the implementation of more-informed self-care measures, adherence to treatments and healthier diet and life-style habits.^31^ Moreover, in our study population, with a mean age about 70 years old in 2012, marked class and gender based inequalities existed in educational attainment, since the accessibility to higher education was limited in families with low economic status, especially for daughters. Further, some studies suggest that adults with lower SES and females, more generally, are less likely to receive preventive treatments for CVD.^12^

Interestingly, the slightly higher SES effect observed in education for females compared with males for CVD was not observed when income is considered (RRR = 0.99; 95%CI: 0.84, 1.16). The absence of a relevant sex-dependent effect for income are in line with the aforementioned recent meta-analysis in general population (RRR = 1.06; 95%CI: 0.85, 1.33),^14^ and also in patients with diabetes.^22^ The discrepancy between education and income indicators could be due to the fact that education level, which usually determines a person’s posterior occupation and income,^32^ could be a more stable proxy of lifetime socioeconomic status than an income measurement,^8^ and therefore, more sensible to detect sex-dependent SES inequalities.

Regarding immigrant status, males that were immigrants showed lower CVD risk than the Spanish patients in our cohort. The “protective” effect of being immigrant has already been observed and attributed to the “healthy immigrant effect”^19^^,^^33^ and also to the fact that immigrants are more likely to be lost to follow-up.^34^ Besides, the CVD event risk in the local-born reference population can also be determinant in the migrant relative risk.^35^ In our cohort there were more males coming from Africa (Morocco) and Europe (Portugal and Bulgaria) than females and more females coming from America (Ecuador and Colombia) than males. Differences in the country of origin might partially explain the lower CVD risk found only for male immigrants compared with female immigrants, but further research is needed to disentangle this aspect.

Noteworthy, when analyzing stroke and CHD separately, magnitudes and especially directions of the RRRs for stroke were in the same line to that observed for global CVD events, but not for CHD risk. These differences were not modified after adjusting for life-style factors, neither adjusting for other traditional risk factors such as HTA or dyslipidemia (data not shown). For education, these results are in contrast to those obtained in the meta-analysis conducted in the general population,^12^ which found similar RRR magnitudes between CHD and CVD, but not between stroke and CVD. In individuals with diabetes, Gnavi et al.^21^ found that education was associated with stroke-related mortality only among males, whereas we observed a significant association in both sexes (higher among females). When income is considered, the HR for CHD in our study was higher among females than among males (RRR = 1.63; 95%CI: 0.82, 3.25). This finding aligns with other studies in general population,^12^^,^^36^ but contrasts with the results reported by Falkentoft et al.^22^ in patients with diabetes, which suggested a higher effect of income on CHD among males than females. Discrepancies between studies could be due to different exposure or outcome definitions, population characteristics, different risk factor adjustments, or other contextual factors.

The slightly sex-dependent effects observed for different SES indicators on CHD or stroke in our study may be due to differences between males and females in hormonal profiles or lifestyle factors, in the distribution of unknown risk factors for each disease or in the response to stress and other environmental factors, which might be more associated with some specific outcomes and may be better captured for one indicator than another. Our finding regarding the higher effect of income observed in females compared with males for CHD (HR = 2.2 vs. 1.3) could be due to an intensification of the pathway linking financial difficulties and psychological stress with specific CHD events in females,^37^ as well as to potential disadvantages in diagnosis, treatment, and prognosis of CHD in females that could be more related with income than with education.^12^^,^^38^

This study has several limitations. First, people with undiagnosed T2D and those exclusively using private health institutions were not included, which may lead to selection bias. Similarly, people who have moved to another region but have not reported this may lead to information bias, especially for the immigrant population, but we expect the proportion of patients in these groups to be small. Second, information regarding lifestyle factors may be partially underreported or outdated and might not coincide with the real baseline status for some patients and third, we have not accounted for the effect of diabetes severity/complications or treatment adherence in the present study. Nevertheless, we consider that these results could be generalized to other regions in Spain and other European southern countries with the similar economic and sociocultural contexts.

Despite the demonstrated association between SES, diabetes and CVD risk, there is an absence of social indicators in clinical decision support systems and in traditional CVD prediction tools,^39^ which might contribute to sustaining these health inequalities. Promisingly, steps are being taken to solve this problem and more evidence supports the fact that integrating socioeconomic indicators into electronic health records could facilitate the prediction of risk, health care utilization and health outcomes.^40^

In conclusion, this population-based study found sex-dependent SES inequalities in the risk of suffering a CVD event, among patients with T2D, having a higher magnitude among females than males when education level is considered. The inclusion of sex-specific SES indicators to identify patients with a high risk of suffering a CVD event and to design tailored preventive and management strategies are likely to have a direct beneficial impact on diabetes-related morbidity and mortality.

## Supplementary Material

ckae048_Supplementary_Data

## Data Availability

Data requests will be considered after the approval of the research ethic committee from the solicitor institution and also from Osasunbidea and NASTAT institutions, who are responsible of the clinical information and the population information, respectively. Requests for collaborative studies are welcome but will still need the abovementioned approval. Key Points Socioeconomic factors may influence differently on cardiovascular events in women and men with type 2 diabetes. Research in sex-dependent relationship between socioeconomic indicators and CVD outcomes in patients with type-2 diabetes is scarce. Both education level and annual income are associated with increased CVD risk in women and men with type 2 diabetes. The magnitude of the association between education level and CVD risk is slightly higher among females than males. These results may help to design more tailored preventive and management strategies aimed to reduce CVD risk in patients with types 2 diabetes.
