# Supplementary material for: Sex-dependent effect of socioeconomic status on cardiovascular event risk in a population-based cohort of patients with type 2 diabetes
Source: Eur J Public Health. 2024 Mar 14;34(3):441–8. doi: 10.1093/eurpub/ckae048 (PMC11161156; doi:10.1093/eurpub/ckae048)
Supplement: ckae048_Supplementary_Data [file ckae048_supplementary_data.docx]

# SUPPLEMENTARY MATERIAL

# Sex-dependent effect of Socioeconomic Status on Cardiovascular Event Risk in a Population-Based Cohort of Patients with Type 2 Diabetes

Mónica Enguita-Germán^1^, Ibai Tamayo^2^, Julián Librero^3^, Asier Ballesteros-Domínguez^4^, Ignacio Oscoz-Villanueva^5^, Arkaitz Galbete^6^, Laura Arnedo^7^, Koldo Cambra^8^, Javier Gorricho^9^, Conchi Moreno-Iribas^10^, Eduardo Millán-Ortuondo^11^, and Berta Ibáñez-Beroiz^12^*

**Table S1.** Outcome definition codes

| ***Fatal cardiovascular disease– cause specific mortality due to any of the following:*** | | |
| --- | --- | --- |
| *Endpoints included* | *ICD10-codes* | *ICD9-codes* |
| Hypertensive disease | I10-16 | 401 – 405 |
| Ischemic heart disease | I20-25 | 410 - 414 |
| Arrhythmias, heart failure | I46-52 | 426 - 429 |
| Cerebrovascular disease | I60-69 | 430 - 438 |
| Atherosclerosis/AAA | I70-73 | 440 - 443 |
| Sudden death and death within 24h of symptom onset | R96.0-96.1 | 798.1 , 798.2 |
|  |  |  |
| Endpoints excluded from the above endpoint: | | |
| Myocarditis, unspecified | I51.4 | 426.7 |
| Subarachnoid haemorrhage | I60 | 429 |
| Subdural haemorrhage | I62 | 430 |
| Cerebral aneurysm | I67.1 | 432.1 |
| Cerebral arteritis | I68.2 | 437.3 |
| Moyamoya | I67.5 | 437.4 |
|  |  |  |
| ***Non-fatal cardiovascular disease*** | | |
| Non-fatal myocardial infarction | I21-I23 | *410* |
| Non-fatal stroke | I60-69 | *430-438* |
|  |  |  |
| *Excluded from the non-fatal stroke endpoint:* | | |
| Subarachnoid hemorrhage | I60 | 429 |
| Subdural hemorrhage | I62 | 430 |
| Cerebral aneurysm | I67.1 | 432.1 |
| Cerebral arteritis | I68.2 | 437.3 |
| Moyamoya | I67.5 | 437.4 |

**Table S2.** Baseline characteristics of the Type 2 Diabetes cohort according to education level and sex

| **Variable** | **Levels** | **Males** | | |  | **Females** | | |
| --- | --- | --- | --- | --- | --- | --- | --- | --- |
|  |  | **Without studies** | **Primary School** | **HS-University** |  | **Without studies** | **Primary School** | **HS-University** |
| **General characteristics** N (%) | | 3611 (26.9) | 7183 (53.5) | 2632 (19.6) |  | 4367 (38.9) | 5881 (52.4) | 976 (8.7) |
| Age | Mean (SD) | 68.9 (12.5) | 64.9 (11.6) | 60.9 (11.3) |  | 73.5 (12.4) | 69.6 (12.2) | 60.8 (14.3) |
| Age group | <60 years | 758 (21.0) | 2247 (31.3) | 1174 (44.6) |  | 550 (12.6) | 1182 (20.1) | 464 (47.5) |
|  | 60-69 years | 955 (26.4) | 2438 (33.9) | 887 (33.7) |  | 822 (18.8) | 1665 (28.3) | 229 (23.5) |
|  | 70-79 years | 1154 (32.0) | 1700 (23.7) | 435 (16.5) |  | 1471 (33.7) | 1667 (28.3) | 181 (18.5) |
|  | ≥80 years | 744 (20.6) | 798 (11.1) | 136 (5.2) |  | 1524 (34.9) | 1367 (23.2) | 102 (10.5) |
| Smoking status | Non-smoker | 1305 (36.1) | 2210 (30.8) | 755 (28.7) |  | 3522 (80.7) | 4164 (70.8) | 469 (48.1) |
|  | Ex-smoker | 1009 (27.9) | 2049 (28.5) | 749 (28.5) |  | 155 (3.5) | 412 (7.0) | 131 (13.4) |
|  | Smoker | 836 (23.2) | 1810 (25.2) | 576 (21.9) |  | 215 (4.9) | 547 (9.3) | 147 (15.1) |
|  | NA | 461 (12.8) | 1114 (15.5) | 552 (21.0) |  | 475 (10.9) | 758 (12.9) | 229 (23.5) |
| Physical activity | Inactive | 246 (6.8) | 424 (5.9) | 165 (6.3) |  | 559 (12.8) | 584 (9.9) | 78 (8.0) |
|  | Partially active | 790 (21.9) | 1412 (19.7) | 461 (17.5) |  | 1320 (30.2) | 1640 (27.9) | 206 (21.1) |
|  | Active | 1894 (52.5) | 3734 (52.0) | 1184 (45.0) |  | 1749 (40.1) | 2508 (42.6) | 349 (35.8) |
|  | NA | 681 (18.9) | 1613 (22.5) | 822 (31.2) |  | 739 (16.9) | 1149 (19.5) | 343 (35.1) |
| Duration T2D | Median (IQR) | 7.0 [4.0, 10.0] | 7.0 [3.0, 10.0] | 6.0 [3.0, 9.2] |  | 7.0 [4.0, 12.0] | 7.0 [4.0, 11.0] | 5.0 [3.0, 9.0] |
| aCharlson group | 1-2 | 2332 (64.6) | 4953 (69.0) | 1899 (72.2) |  | 3169 (72.6) | 4314 (73.4) | 745 (76.3) |
|  | ≥3 | 1279 (35.4) | 2230 (31.0) | 733 (27.8) |  | 1198 (27.4) | 1567 (26.6) | 231 (23.7) |
| **Patient level SES characteristics** | |  |  |  |  |  |  |  |
| Income | <18000€ | 2646 (73.3) | 4268 (59.4) | 1044 (39.7) |  | 3933 (90.1) | 4762 (81.0) | 528 (54.1) |
|  | ≥18000€ | 965 (26.7) | 2915 (40.6) | 1588 (60.3) |  | 434 (9.9) | 1119 (19.0) | 448 (45.9) |
| Immigrant | No | 3360 (93.0) | 6941 (96.6) | 2497 (94.9) |  | 4061 (93.0) | 5627 (95.7) | 875 (89.7) |
|  | Yes | 251 (7.0) | 242 (3.4) | 135 (5.1) |  | 306 (7.0) | 254 (4.3) | 101 (10.3) |

HS-University: High-School or University.

**Table S3.** Baseline characteristics of the Type 2 Diabetes cohort according to income and sex

| **Variable** | **Levels** | | **Males** | | **Females** | |
| --- | --- | --- | --- | --- | --- | --- |
|  |  | | <18000€ | ≥18000€ | <18000€ | ≥18000€ |
| **General characteristics** | | **N (%)** | 7958 (59.3) | 5468 (40.7) | 9223 (82.2) | 2001 (17.8) |
| Age | Mean (SD) | | 66.1 (13.1) | 63.9 (10.3) | 71.4 (13.0) | 65.9 (11.6) |
| Age group | <60 years | | 2490 (31.3) | 1689 (30.9) | 1665 (18.0) | 531 (26.5) |
|  | 60-69 years | | 2000 (25.1) | 2280 (41.7) | 1959 (21.2) | 757 (37.8) |
|  | 70-79 years | | 2152 (27.0) | 1137 (20.8) | 2859 (31.0) | 460 (23.0) |
|  | ≥80 years | | 1316 (16.5) | 362 (6.6) | 2740 (29.7) | 253 (12.6) |
| Smoking status | Non-smoker | | 2557 (32.1) | 1713 (31.3) | 6881 (74.6) | 1274 (63.7) |
|  | Ex-smoker | | 2171 (27.3) | 1636 (29.9) | 489 (5.3) | 209 (10.4) |
|  | Smoker | | 1984 (24.9) | 1238 (22.6) | 688 (7.5) | 221 (11.0) |
|  | NA | | 1246 (15.7) | 881 (16.1) | 1165 (12.6) | 297 (14.8) |
| Physical activity | Inactive | | 569 (7.2) | 266 (4.9) | 1079 (11.7) | 142 (7.1) |
|  | Partially active | | 1743 (21.9) | 920 (16.8) | 2694 (29.2) | 472 (23.6) |
|  | Active | | 3867 (48.6) | 2945 (53.9) | 3688 (40.0) | 918 (45.9) |
|  | NA | | 1779 (22.4) | 1337 (24.5) | 1762 (19.1) | 469 (23.4) |
| Duration T2D | Median (IQR) | | 6.0 [3.0, 10.0] | 7.0 [3.0, 10.0] | 7.0 [4.0, 11.0] | 7.0 [3.0, 10.0] |
| aCharlson group | 1-2 | | 5340 (67.1) | 3844 (70.3) | 6730 (73.0) | 1498 (74.9) |
|  | ≥3 | | 2618 (32.9) | 1624 (29.7) | 2493 (27.0) | 503 (25.1) |
| **SES characteristics** | | |  |  |  |  |
| Education Level | Without studies | | 2646 (33.2) | 965 (17.6) | 3933 (42.6) | 434 (21.7) |
|  | Primary School | | 4268 (53.6) | 2915 (53.3) | 4764 (51.6) | 1119 (55.9) |
|  | High School - University | | 1044 (13.1) | 1588 (29.0) | 528 (5.7) | 448 (22.4) |
| Immigrant | No | | 7446 (93.6) | 5352 (97.9) | 8616 (93.4) | 1949 (97.4) |
|  | Yes | | 512 (6.4) | 116 (2.1) | 609 (6.6) | 52 (2.6) |

**Table S4.** Baseline characteristics of the Type 2 Diabetes cohort according to immigration status and sex

| **Variable** | **Levels** | **Males** | |  | **Females** | | |
| --- | --- | --- | --- | --- | --- | --- | --- |
|  |  | **Spanish** | **Immigrant** |  | **Spanish** | **Immigrant** | |
| **General characteristics** N (%) | | 12798 (95.3) | 628 (4.7) |  | 10563 (94.1) | 661 (5.9) |  |
| Age | Mean (SD) | 65.8 (11.8) | 52.1 (10.6) |  | 71.4 (12.3) | 54.6 (12.8) | |
| Age group | <60 years | 3689 (28.8) | 490 (78.0) |  | 1730 (16.4) | 466 (70.5) | |
|  | 60-69 years | 4188 (32.7) | 92 (14.6) |  | 2601 (24.6) | 115 (17.4) | |
|  | 70-79 years | 3253 (25.4) | 36 (5.7) |  | 3266 (30.9) | 53 (8.0) | |
|  | ≥80 years | 1668 (13.0) | 10 (1.6) |  | 2966 (28.1) | 27 (4.1) | |
| Smoking status | Non-smoker | 4045 (31.6) | 225 (35.8) |  | 7736 (73.2) | 419 (63.4) | |
|  | Ex-smoker | 3693 (28.9) | 114 (18.2) |  | 653 (6.2) | 45 (6.8) | |
|  | Smoker | 3086 (24.1) | 136 (21.7) |  | 836 (7.9) | 73 (11.0) | |
|  | NA | 1974 (15.4) | 153 (24.4) |  | 1338 (12.7) | 124 (18.8) | |
| Physical activity | Inactive | 783 (6.1) | 52 (8.3) |  | 1168 (11.1) | 53 (8.0) | |
|  | Partially active | 2540 (19.8) | 123 (19.6) |  | 2991 (28.3) | 175 (26.5) | |
|  | Active | 6558 (51.2) | 254 (40.4) |  | 4342 (41.1) | 264 (39.9) | |
|  | NA | 2917 (22.8) | 199 (31.7) |  | 2062 (19.5) | 169 (25.6) | |
| Duration T2D | Median (IQR) | 7.0 [3.0, 10.0] | 4.0 [2.0, 6.0] |  | 7.0 [4.0, 11.0] | 4.0 [2.0, 7.0] | |
| aCharlson group | 1-2 | 8627 (67.4) | 557 (88.7) |  | 7657 (72.5) | 571 (86.4) | |
|  | ≥3 | 4171 (32.6) | 71 (11.3) |  | 2906 (27.5) | 90 (13.6) | |
| **SES characteristics** | |  |  |  |  |  |  |
| Education Level | Without studies | 2497 (19.5) | 135 (21.5) |  | 875 (8.3) | 101 (15.3) | |
|  | Primary School | 6941 (54.2) | 242 (38.5) |  | 5627 (53.3) | 254 (38.4) | |
|  | High School - University | 3360 (26.3) | 251 (40.0) |  | 4061 (38.4) | 306 (46.3) | |
| Income | <18000€ | 5352 (41.8) | 116 (18.5) |  | 1949 (18.5) | 52 (7.9) | |
|  | ≥18000€ | 7446 (58.2) | 512 (81.5) |  | 8614 (81.5) | 609 (92.1) | |

**Table S5.** Adjusted Fine-Gray competing risk HRs (95%CI) for Education level on CVD, stroke and CHD outcomes

|  |  | **CVD event** | | | **Stroke** | | | **CHD** | | |
| --- | --- | --- | --- | --- | --- | --- | --- | --- | --- | --- |
| **Model** |  | **Males** | **Females** | **Females/Males** | **Males** | **Females** | **Females/Males** | **Males** | **Females** | **Females/Males** |
| Variable |  | HR (95CI) | HR (95CI) | RRR (95CI) |  |  | RRR (95CI) |  |  | RRR (95CI) |
| **Min-adjusted** |  |  |  |  |  |  |  |  |  |  |
| Age | <60 years | Reference | Reference |  | Reference | Reference |  | Reference | Reference |  |
|  | 60-69 years | 1.41(1.07-1.86) | 1.26 (0.90-1.75) |  | 1.92 (1.45-2.54) | 1.21 (0.74-1.98) |  | 0.90 (0.59-1.38) | 1.27 (0.96-1.68) |  |
|  | 70-79 years | 1.95 (1.59-2.39) | 2.24 (1.66-3.02) |  | 2.56 (2.02-3.26) | 2.16 (1.53-3.05) |  | 1.07 (0.82-1.42) | 1.74 (1.08-2.81) |  |
|  | ≥80 years | 3.09 (2.28-4.18) | 5.59 (4.53-6.91) |  | 3.60 (2.32-5.59) | 4.40 (3.50-5.54) |  | 1.17 (0.76-1.82) | 3.06 (2.05-4.56) |  |
| Duration T2D | 10-years | 1.21 (1.15-1.28) | 1.27 (1.21-1.32) |  | 1.12 (1.05-1.19) | 1.33 (1.26-1.39) |  | 1.39 (1.23-1.56) | 1.41 (1.27-1.57) |  |
| aCharlson group | 1-2 | Reference | Reference |  | Reference | Reference |  | Reference | Reference |  |
|  | ≥3 | 1.81 (1.53-2.15) | 2.06 (1.86-2.29) |  | 2.13 (1.80-2.51) | 2.25 (1.99-2.54) |  | 1.79 (1.36-2.34) | 2.20 (1.73-2.79) |  |
| Education Level | HS-University | Reference | Reference |  | Reference | Reference |  | Reference | Reference |  |
|  | Primary School | 1.15 (0.95-1.38) | 1.33 (0.94-1.88) | 1.16 (0.78-1.71) | 1.26 (1.07-1.49) | 1.45 (1.03-2.04) | 1.15 (0.79-1.68) | 1.02 (0.83-1.27) | 1.00 (0.56-1.76) | 0.98 (0.53-1.81) |
|  | Without studies | 1.24 (1.09-1.41) | 1.50 (1.09-2.06) | 1.21 (0.86-1.71) | 1.21 (0.97-1.51) | 1.46 (0.94-2.26) | 1.21 (0.74-1.97) | 1.28 (1.12-1.45) | 1.25 (0.62-2.49) | 0.98 (0.48-1.98) |
| **Max-adjusted** |  |  |  |  |  |  |  |  |  |  |
| Education Level | HS-University | Reference | Reference |  | Reference | Reference |  | Reference | Reference |  |
|  | Primary School | 1.14 (0.94-1.38) | 1.37 (0.96-1.95) | 1.20 (0.80-1.80) | 1.28 (1.07-1.53) | 1.47 (1.03-2.10) | 1.15 (0.77-1.71) | 0.99 (0.81-1.22) | 0.99 (0.56-1.77) | 1.00 (0.54-1.84) |
|  | Without studies | 1.23 (1.10-1.38) | 1.52 (1.10-2.10) | 1.24 (0.88-1.74) | 1.22 (1.00-1.49) | 1.46 (0.93-2.29) | 1.20 (0.73-1.96) | 1.22 (1.07-1.40) | 1.25 (0.61-2.56) | 1.02 (0.49-2.13) |

HS-University: High School – University. The min-adjusted model included the individual-level SES indicator (level of education, income or immigrant status), the annual mean income of the area as a clustering variable and age, duration of diabetes and aCharlson score as potential confounders. The max-adjusted model included also Smoking status and Physical activity (estimates for all variables not shown for simplicity)

**Table S6.** Adjusted Fine-Gray competing risk HRs (95%CI) for Income level on CVD, stroke and CHD outcomes

|  |  | **CVD event** | | | **Stroke** | | | **CHD** | | |
| --- | --- | --- | --- | --- | --- | --- | --- | --- | --- | --- |
| **Model** |  | **Males** | **Females** | **Females/Males** | **Males** | **Females** | **Females/Males** | **Males** | **Females** | **Females/Males** |
| Variable |  | HR (95CI) | HR (95CI) | RRR (95CI) | HR (95CI) | HR (95CI) | RRR (95CI) | HR (95CI) | HR (95CI) | RRR (95CI) |
| **Min-adjusted** |  |  |  |  |  |  |  |  |  |  |
| Age | <60 years | Reference | Reference |  | Reference | Reference |  | Reference | Reference |  |
|  | 60-69 years | 1.50 (1.14-1.97) | 1.32 (0.97-1.80) |  | 2.04 (1.56-2.68) | 1.27 (0.80-2.04) |  | 0.95 (0.61-1.46) | 1.32 (0.98-1.78) |  |
|  | 70-79 years | 1.97 (1.64-2.35) | 2.31 (1.72-3.10) |  | 2.55 (2.07-3.15) | 2.23 (1.57-3.16) |  | 1.10 (0.86-1.42) | 1.71 (0.97-3.02) |  |
|  | ≥80 years | 3.03 (2.34-3.92) | 5.73 (4.65-7.07) |  | 3.44 (2.31-5.11) | 4.51 (3.53-5.77) |  | 1.19 (0.81-1.75) | 2.94 (1.91-4.54) |  |
| Duration T2D | 10-years | 1.22 (1.15-1.29) | 1.27 (1.21-1.32) |  | 1.13 (1.07-1.20) | 1.33 (1.26-1.40) |  | 1.39 (1.23-1.57) | 1.40 (1.27-1.55) |  |
| aCharlson group | 1-2 | Reference | Reference |  | Reference | Reference |  | Reference | Reference |  |
|  | ≥3 | 1.80 (1.52-2.14) | 2.06 (1.85-2.29) |  | 2.11 (1.78-2.51) | 2.24 (1.98-2.53) |  | 1.78 (1.36-2.33) | 2.19 (1.71-2.81) |  |
| Income | ≥18000€ | Reference | Reference |  | Reference | Reference |  | Reference | Reference |  |
|  | <18000€ | 1.44 (1.29-1.59) | 1.42 (1.26-1.60) | 0.99 (0.84-1.16) | 1.53 (1.39-1.68) | 1.24 (0.90-1.72) | 0.81 (0.58-1.14) | 1.34 (1.04-1.72) | 2.19 (1.15-4.14) | 1.63 (0.82-3.25) |
| **Max-adjusted** |  |  |  |  |  |  |  |  |  |  |
| Income | ≥18000€ | Reference | Reference |  | Reference | Reference |  | Reference | Reference |  |
|  | <18000€ | 1.39 (1.25-1.54) | 1.40 (1.24-1.57) | 1.01 (0.86-1.18) | 1.49 (1.37-1.62) | 1.23 (0.89-1.70) | 0.83 (0.59-1.15) | 1.28 (1.00-1.64) | 2.14 (1.13-4.08) | 1.67 (0.84-3.33) |

HS-University: High School – University. The min-adjusted model included the individual-level SES indicator (level of education, income or immigrant status), the annual mean income of the area as a clustering variable and age, duration of diabetes and aCharlson score as potential confounders. The max-adjusted model included also Smoking status and Physical activity (estimates for all variables not shown for simplicity).

**Table S7.** Adjusted Fine-Gray competing risk HRs (95%CI) for Immigrant status on CVD, stroke and CHD outcomes

| **Model** |  | **CVD event** | | | **Stroke** | | | **CHD** | | |
| --- | --- | --- | --- | --- | --- | --- | --- | --- | --- | --- |
|  |  | **Males** | **Females** | **Females/Males** | **Males** | **Females** | **Females/Males** | **Males** | **Females** | **Females/Males** |
| Variable |  | HR (95CI) | HR (95CI) | RRR (95CI) | HR (95CI) | HR (95CI) | RRR (95CI) | HR (95CI) | HR (95CI) | RRR (95CI) |
| **Min-adjusted** |  |  |  |  |  |  |  |  |  |  |
| Age | <60 years | Reference | Reference |  | Reference | Reference |  | Reference | Reference |  |
|  | 60-69 years | 1.41 (1.09-1.83) | 1.33 (0.97-1.83) |  | 1.92 (1.48-2.49) | 1.36 (0.82-2.24) |  | 0.91 (0.60-1.39) | 1.26 (0.96-1.66) |  |
|  | 70-79 years | 1.98 (1.65-2.39) | 2.44 (1.73-3.44) |  | 2.59 (2.12-3.17) | 2.47 (1.68-3.62) |  | 1.12 (0.85-1.48) | 1.78 (1.02-3.09) |  |
|  | ≥80 years | 3.19 (2.44-4.17) | 6.17 (4.79-7.95) |  | 3.66 (2.49-5.39) | 5.07 (3.80-6.78) |  | 1.26 (0.84-1.88) | 3.16 (2.07-4.83) |  |
| Duration T2D | 10-years | 1.21 (1.15-1.27) | 1.27 (1.22-1.32) |  | 1.12 (1.06-1.20) | 1.33 (1.27-1.40) |  | 1.38 (1.23-1.56) | 1.41 (1.28-1.55) |  |
| aCharlson group | 1-2 | Reference | Reference |  | Reference | Reference |  | Reference | Reference |  |
|  | ≥3 | 1.80 (1.53-2.13) | 2.06 (1.85-2.28) |  | 2.12 (1.80-2.50) | 2.25 (1.99-2.54) |  | 1.79 (1.36-2.34) | 2.18 (1.72-2.77) |  |
| Immigrant | No | Reference | Reference |  | Reference | Reference |  | Reference | Reference |  |
|  | Yes | 0.81 (0.66-0.99) | 1.13 (0.68-1.87) | 1.4 (0.81-2.41) | 0.83 (0.47-1.46) | 1.53 (0.74-3.17) | 1.84 (0.73-4.64) | 0.99 (0.67-1.47) | 0.81 (0.50-1.30) | 0.82 (0.44-1.52) |
| **Max-adjusted** |  |  |  |  |  |  |  |  |  |  |
| Immigrant | No | Reference | Reference |  | Reference | Reference |  | Reference | Reference |  |
|  | Yes | 0.83 (0.67-1.03) | 1.17 (0.73-1.87) | 1.41 (0.84-2.36) | 0.82 (0.46-1.45) | 1.56 (0.77-3.15) | 1.9 (0.77-4.72) | 1.04 (0.71-1.53) | 0.87 (0.56-1.35) | 0.84 (0.47-1.50) |

HS-University: High School – University. The min-adjusted model included the individual-level SES indicator (level of education, income or immigrant status), the annual mean income of the area as a clustering variable and age, duration of diabetes and aCharlson score as potential confounders. The max-adjusted model included also Smoking status and Physical activity (estimates for all variables not shown for simplicity)

.

**Table S8.** Adjusted Fine-Gray competing risk HRs (95%CI) of CVD event for all SES indicators together (Education level, Income and immigrant status)

| **Model** |  | **CVD event** | | | **Stroke** | | | **CHD** | | |
| --- | --- | --- | --- | --- | --- | --- | --- | --- | --- | --- |
|  |  | **Males** | **Females** | **Females/Males** | **Males** | **Females** | **Females/Males** | **Males** | **Females** | **Females/Males** |
|  |  | HR (95CI) | HR (95CI) | RRR (95CI) | HR (95CI) | HR (95CI) | RRR (95CI) | HR (95CI) | HR (95CI) | RRR (95CI) |
| **Min-adjusted** |  |  |  |  |  |  |  |  |  |  |
| Education Level | HS-University | Reference | Reference |  | Reference | Reference |  | Reference | Reference |  |
|  | Primary School | 1.06 (0.86-1.32) | 1.23 (0.87-1.73) | 1.16 (0.77-1.74) | 1.16 (0.96-1.39) | 1.40 (0.97-2.01) | 1.21 (0.80-1.82) | 0.97 (0.77-1.22) | 0.82 (0.43-1.58) | 0.85 (0.42-1.69) |
|  | Without studies | 1.12 (0.96-1.31) | 1.37 (1.01-1.85) | 1.22 (0.87-1.72) | 1.07 (0.86-1.33) | 1.38 (0.88-2.18) | 1.29 (0.78-2.13) | 1.18 (1.01-1.38) | 1.01 (0.48-2.11) | 0.86 (0.40-1.82) |
| Income | ≥18000€ | Reference | Reference |  | Reference | Reference |  | Reference | Reference |  |
|  | <18000€ | 1.42 (1.26-1.61) | 1.36 (1.24-1.49) | 0.96 (0.82-1.12) | 1.52 (1.40-1.66) | 1.18 (0.85-1.64) | 0.78 (0.55-1.09) | 1.31 (1.01-1.70) | 2.20 (1.06-4.56) | 1.68 (0.77-3.64) |
| Immigrant | No | Reference | Reference |  |  |  |  |  |  |  |
|  | Yes | 0.75 (0.62-0.90) | 1.07 (0.66-1.75) | 1.43 (0.85-2.40) | 0.78 (0.45-1.33) | 1.51 (0.73-3.10) | 1.94 (0.78-4.78) | 0.90 (0.59-1.37) | 0.71 (0.41-1.23) | 0.79 (0.39-1.58) |
| **Max-adjusted** |  |  |  |  |  |  |  |  |  |  |
| Education Level | HS-University | Reference | Reference |  | Reference | Reference |  | Reference | Reference |  |
|  | Primary School | 1.07 (0.87-1.32) | 1.27 (0.89-1.81) | 1.19 (0.79-1.79) | 1.18 (0.97-1.43) | 1.42 (0.97-2.07) | 1.20 (0.79-1.84) | 0.95 (0.76-1.18) | 0.83 (0.44-1.58) | 0.87 (0.44-1.72) |
|  | Without studies | 1.12 (0.97-1.30) | 1.40 (1.02-1.91) | 1.25 (0.88-1.77) | 1.08 (0.89-1.32) | 1.39 (0.87-2.22) | 1.29 (0.77-2.14) | 1.14 (0.96-1.36) | 1.03 (0.49-2.18) | 0.90 (0.42-1.94) |
| Income | ≥18000€ | Reference | Reference |  | Reference | Reference |  | Reference | Reference |  |
|  | <18000€ | 1.37 (1.21-1.55) | 1.33 (1.22-1.45) | 0.97 (0.83-1.13) | 1.48 (1.37-1.60) | 1.17 (0.84-1.62) | 0.79 (0.56-1.11) | 1.26 (0.97-1.63) | 2.15 (1.04-4.43) | 1.71 (0.79-3.68) |
| Immigrant | No | Reference | Reference |  | Reference | Reference |  | Reference | Reference |  |
|  | Yes | 0.77 (0.63-0.94) | 1.12 (0.71-1.76) | 1.45 (0.89-2.39) | 0.77 (0.45-1.34) | 1.54 (0.77-3.09) | 2.00 (0.83-4.84) | 0.96 (0.63-1.45) | 0.77 (0.46-1.27) | 0.8 (0.42-1.55) |

**Abbreviations:** HS-University: High School – University. The min-adjusted model included the individual-level SES indicator (level of education, income or immigrant status), the annual mean income of the area as a clustering variable and age, duration of diabetes and aCharlson score as potential confounders. The max-adjusted model included also Smoking status and Physical activity (estimates for all variables not shown for simplicity)
